# Doctors vs. Tailors

**Published:** 1857-01

**Authors:** 


### DOCTORS *vs.* TAILORS,

And the doctor triumphant! In a recent criminal trial, a merchant tailor testified that he would not trust the prisoner, because he knew that any man must fail, who in a single year would order to be made in the best manner and out of the best material seven coats, fifteen vests, and twenty-five pair of pantaloons.

The doctor, homeopathic in pills but *all*-pathic in charges, his bill averaging six hundred dollars a year, stated that he drew more money than he was entitled to, "because he seemed to be living too fast, and I intended to get my bills about square."

